# Immunohistochemical Examination of Trophoblast Syncytialization during Early Placentation in Sheep

**DOI:** 10.3390/ijms20184530

**Published:** 2019-09-13

**Authors:** Heewon Seo, Fuller W. Bazer, Robert C. Burghardt, Greg A. Johnson

**Affiliations:** 1 Department of Veterinary Integrative Biosciences, Texas A&M University, College Station, TX 77843, USA; hseo@cvm.tamu.edu (H.S.); rburghardt@cvm.tamu.edu (R.C.B.); 2 Department of Animal Science, Texas A&M University, College Station, TX 77843, USA; fbazer@cvm.tamu.edu

**Keywords:** trophoblast, uterine luminal epithelium, syncytialization, sheep

## Abstract

During the peri-implantation period, multinucleated syncytia are formed in the sheep placenta. For over 20 years the scientific consensus has been that during trophoblast syncytialization in sheep, binucleate trophoblast giant cells (BNCs) differentiate from mononuclear trophoblast cells, and individual BNCs fuse with individual luminal epithelial (LE) cells to form trinucleate cells. These trophoblast–LE syncytial plaques then grow through continued BNC migration and fusion. Therefore, LE cells are thought to be incorporated into syncytial plaques. However, these ideas were based on electron microscopy studies, without benefit of molecular markers for BNC and LE cells to support conclusions. The aim of this study was to observe interactions between BNCs and uterine LE cells using immunohistochemical localization for molecular markers for BNCs and uterine LE cells. We performed immunofluorescence staining, laser capture microdissection, and TUNEL staining on the uterine–placental tissues of sheep during early placentation. We observed: (1) syncytial cells containing more than two nuclei within the trophoblast cell layer; (2) depolarized LE cells that express caspase 3 and stain positively for TUNEL; (3) engulfment of caspase 3-positive LE cells by trophoblast giant cells (TGCs) and empty spaces within the LE layer at sites of implantation; (4) rapid enlargement of syncytial plaques; and (5) E-cadherin and TUNEL-positive cells within the uterine stroma underlying degenerating LE was coincident with accumulation of CD45-positive cells at these sites. These data suggest that during early placentation: (1) fusion between trophoblasts is not limited to the formation of BNCs, and the term ‘trophoblast giant cell (TGC)’ may be appropriate; (2) LE cells undergo apoptosis; (3) apoptotic LE cells are eliminated by TGCs; (4) fusion is not limited to the incorporation of new BNCs but involves the lateral fusion between growing syncytial plaques; and (5) TGCs carry apoptotic LE cells away from the uterine–placental interface for elimination by immune cells within the stroma. These data indicate that uterine LE cells are not incorporated into syncytial plaques, but are engulfed and eliminated, and that early placentation in sheep is more similar to early placentation in humans than is currently understood in that both develop mononucleated cytotrophoblast and multinucleated syncytiotrophoblast layers of entirely placental origin. The elimination of LE cells by sheep TGCs might provide insights into elimination and penetration of LE cells during human embryo implantation.

## 1. Introduction

The conceptuses of sheep remain free floating within the uterine lumen as they elongate from spherical blastocysts to conceptuses with a filamentous morphology [1]. Sheep embryos enter the uterus on day 3, develop to spherical blastocysts, and, subsequent to hatching from the zona pellucida, transform from spherical to tubular and filamentous conceptuses between days 12 and 15 of pregnancy. The conceptus extra-embryonic membranes extend into the contralateral uterine horn, relative to the ovary with a corpus luteum, between days 16 and 20 of pregnancy [2]. During this period of rapid elongation and differentiation, the mononuclear trophoblast cells of ovine conceptuses secrete interferon tau as the pregnancy recognition signal and attachment of the trophoblast to the uterine LE is initiated [3,4,5]. After firm attachment of the trophoblast to the uterine LE, ruminants, including sheep and cattle, develop synepitheliochorial placentae in which two morphologically and functionally distinct trophoblast cell types are present at the uterine–placental interface of placentomes: the mononucleated trophoblasts and the multinucleated syncytia. These trophoblast cells control the exchange of gases, nutrients, and other factors between the maternal and fetal circulations, protect the fetus against the maternal immune system, and are responsible for the production of many proteins, hormones, and growth factors [1,2].

Initially the mononucleate trophoblast cells constitute the majority of the trophoblast cells; however, between days 14 and 16 of gestation, binucleate trophoblast cells (BNCs) begin to differentiate from the mononuclear trophoblast cells by consecutive nuclear divisions without cytokinesis, also termed mitotic polyploidy [6,7,8]. By day 18, they comprise 15–20% of the trophoblast cells that are apposed to the uterine LE at sites of conceptus implantation [9,10]. The BNCs pass through the tight junctions between adjacent mononucleate trophoblast cells and migrate to the uterine LE for syncytialization with LE cells [7]. In addition, BNCs express placental lactogens and pregnancy-associated glycoproteins (PAGs). The measurement of PAGS in serum can be reliably utilized to assess BNC development, and diagnose pregnancy in both sheep and cattle [11].

The process of syncytia formation in sheep is generally explained by Wooding’s hypothesis (Figure S1) [12]. This hypothesis states that BNCs differentiate from the mononuclear trophoblast cells and migrate and fuse with individual uterine LE cells to form trinucleate syncytial cells, thereby assimilating the LE cells. BNCs continue to develop and migrate to the LE layer and fuse with these growing trophoblast–LE syncytial cells to eventually form extensive syncytial plaques. Therefore, these syncytial plaques are conceptus–maternal hybrid cells that are composed of LE cells and BNCs, and they eventually cover the entire caruncular surface to form the epithelial interface between uterine caruncular and placental cotyledonary tissues within the placentome of sheep. However, this idea was based on electron microscopy studies, without the benefit of molecular markers of BNC and LE to support the conclusion. Therefore, the aim of this study was to perform immunohistochemical localization for molecular markers for BNCs and uterine LE cells, including PAGs, E-cadherin, cytokeratin, and caspase 3, as well as assess apoptosis (TUNEL staining) at the uterine–placental interface to better understand the mechanism for syncytialization during early placentation in sheep.

## 2. Results

### 2.1. Validation of the Efficacy of Observing BNCs and Syncytia during Early Placentation in Sheep

**Figure 1:**
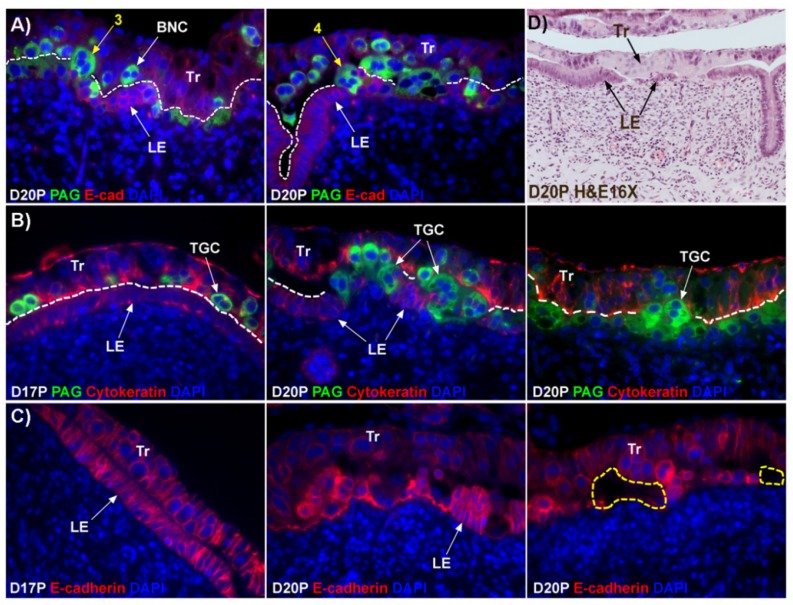
Interaction between TGCs and uterine LE cells at implantation sites in sheep. (**A**) Within the trophoblast layer, there are PAG-stained binucleate cells (BNC) as well as trophoblast cells that contain 3 or 4 nuclei (3 and 4 shown in yellow and termed trophoblast giant cells throughout the manuscript). The dotted line delineates the interface between the trophoblast layer and uterine LE layer. (**B**) Double immunofluorescence staining for PAG and cytokeratin at implantation sites between day 17 and day 20 of pregnancy. Most TGCs are present within the trophoblast layer on day 17 of pregnancy, but on day 20, there is extensive migration of TGCs into the uterine LE cell layer and replacement of mononuclear uterine LE cells by syncytial cells. (**C**) E-cadherin (E-cad; red, stains mononuclear trophoblast and LE) immunostaining at implantation sites revealed the development of gaps/voids (yellow dotted line) in the uterine LE layer. (**D**) H and E staining of a day 20 implantation site in sheep illustrates the absence of sections of uterine LE that are replaced by syncytial cells. Tr, mononuclear trophoblast cells; BNC, binucleate trophoblast cell; TGC, trophoblast giant cell; LE, luminal epithelium; D, day; P, pregnancy. The width of field for the microscopic images of immunofluorescence is 220 μm. The width of the field for the H and E image is 540 μm.

To observe BNC formation within the trophoblast and syncytialization within the uterine LE layer, immunofluorescence staining for PAG and cytokeratin was performed (Figure 1). PAG protein was localized to the binucleate trophoblast cells (BNCs) in the conceptus trophoblast layer on day 17 of pregnancy (Figure S1A). Both mononucleate trophoblast cells and uterine LE cells stained positively for cytokeratin; however, immunostaining for cytokeratin was more intense in the mononucleate trophoblast than was observed in the uterine LE cells on day 17 of pregnancy (Figure S1B). The cytokeratin-positive LE cells were replaced by multinucleated syncytia at implantation sites on day 30 of pregnancy. Cytokeratin was barely detectable in the syncytia (Figure S1C).

### 2.2. Trophoblast Giant Cells (TGCs) that have Two or More Nuclei Are Generated from Mononucleate Trophoblast Cells of Sheep

BNCs and syncytial cells were localized at the uterine–placental interface of sheep by double immunofluorescence to detect the PAGs and E-cadherin (Figure 1A). As predicted, E-cadherin localized to mononuclear trophoblast cells and uterine LE cells, and the PAGs localized to BNCs within the trophoblast layer. PAG-immunostaining was also present in trophoblast cells migrating out of the trophoblast layer that contained three and four nuclei. Since these cells contain more than two nuclei, we use the term, trophoblast giant cells (TGCs) for multinuclear trophoblast cells within the trophoblast layer.

### 2.3. Considerable Syncytialization and Formation of Gaps in the Uterine LE Layer Occurs between Day 17 and Day 20 of Pregnancy in Sheep

To observe interaction between TGCs and LE cells, we performed double immunofluorescence staining for PAGs and cytokeratin. Most of the TGCs were observed within the trophoblast layer on day 17 of pregnancy, but, on day 20 of pregnancy, extensive migration of TGCs into the LE cell layer and replacement of mononuclear uterine LE cells by TGCs was observed (Figure 1B). Fewer nuclei were observed in the syncytialized regions of the LE layer as compared to the number of nuclei observed in regions of intact LE on day 17 of pregnancy.

**Figure 2:**
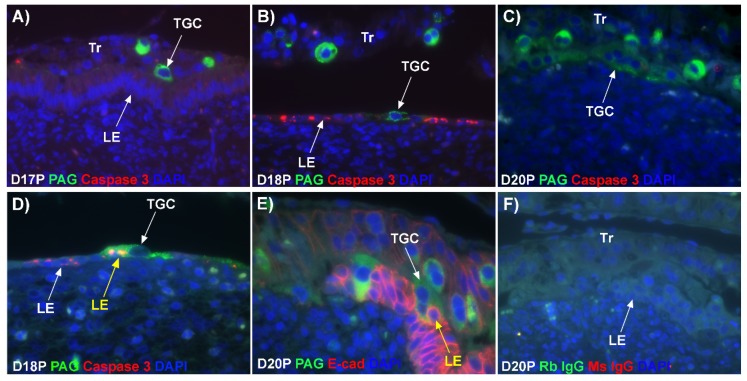
Expression of caspase 3 and internalization of caspase 3-positive LE cells by TGCs at implantation sites of sheep. (**A**–**D**) Double immunofluorescence staining for PAGs (green, stains TGCs) and caspase 3 (red). The yellow arrow indicates a TGC endocytosing caspase 3-positive LE cells. (**E**) Immunofluorescence for PAGs (green, stains TGCs) and E-cadherin (E-cad; red, stains mononucleate Tr and LE). The yellow arrow indicates a TGC appearing to physically engulf an LE cell. The dual labelled rabbit (Rb) and mouse (Ms) IgG control is presented in panel (**F**). Tr, mononuclear trophoblast cells; TGC, trophoblast giant cells; LE, luminal epithelium; D, day; P, pregnancy. The width of field for the microscopic images in panels is 220 μm.

Since fewer nuclei were observed during syncytialization, we questioned whether loss of LE cells occurs during syncytialization. Immunofluorescence staining for E-cadherin showed that LE cells were intact and polarized on day 17 of pregnancy, which is the period of initial attachment of conceptus trophoblast to LE (Figure 1C). By day 18 the LE cells became flattened (see Figure 2B,D), then became depolarized with gaps forming in the LE layer by day 20 at sites of implantation (Figure 1C,D).

### 2.4. Caspase 3 Protein Is Present in Uterine LE Cells and TGCs Internalize Caspase 3-Positive LE Cells during Syncytialization

Since we observed gaps forming in the uterine LE layer, we immunolocalized caspase 3, an executor in the apoptosis cascade, during early placentation in sheep. Immunofluorescence signal for caspase 3 were not above background in the intact LE on day 17 of pregnancy (Figure 2A). However, by day 18, flattened mononuclear LE cells expressed caspase 3 in intermittent regions of the uterine–placental interface, and by day 20 of gestation, LE cells were only infrequently observed and the predominant cell type in the LE layer was the growing syncytial TGCs (Figure 2B,C). In addition, the TGCs appeared to physically engulf the apoptotic LE cells (Figure 2D,E; see LE identified using yellow arrows).

### 2.5. E-Cadherin-Positive Cells Were Detected within the Stroma Underlying the Syncytialized LE

**Figure 3:**
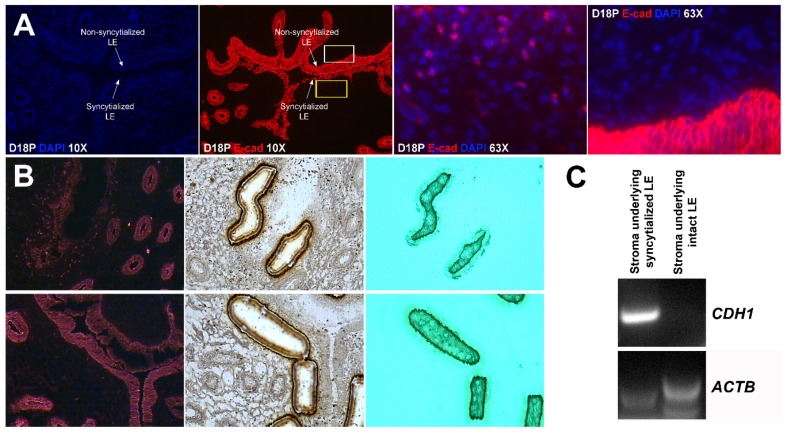
Localization of E-cadherin-positive cells within the uterine stroma during active syncytialization at implantation sites of sheep. (**A**) Immunofluorescence staining for E-cadherin (E-cad, red) in the endometrium on day 18 of pregnancy. The third panel represents the region indicated by the yellow box in the second panel. The fourth panel represents the region indicated by the white box in the second panel. E-cadherin-positive cells are present within the stroma underlying actively syncytializing uterine LE. The width of field for the microscopic image captured at 10× and 63× is 890 and 140 μm, respectively. (**B**) Laser capture microdissection (LCM) used to collect cells within the stroma underlying syncytializing LE (top row) and cells within the stroma underlying intact uterine LE (bottom row). Frozen endometrial tissue sections on day 18 of pregnancy stained with E-cadherin immediately before microdissection (top and bottom rows, first panels). The same tissue section is shown with the missing cells after microdissection (top and bottom rows, middle panels). The LCM cells attached to the CapSure Macro LCM Caps (top and bottom rows, third panels). The width of field for the microscopic image is 631 μm. (**C**) RT-PCR analysis of CDH1 mRNA in the stromal cells captured by LCM. ACTB was used as a positive control. CDH1 mRNA was detected in the LCM cells isolated from stroma underlying actively syncytializing LE.

In order to carefully assess the location of all epithelial cells, in particular those LE cells that appeared to be no longer present in the LE layer, we next performed immunofluorescence localization for E-cadherin in frozen sections of uterine–placental tissues from day 18. We have observed in the past that the antigenicity of many proteins is better maintained in tissue sections frozen in OCT compound than in tissue sections that are paraformaldehyde-fixed and paraffin-embedded, although the paraffin-embedded sections maintain superior histology. A cross-section of endometrium from day 18 of gestation includes intact and non-syncytialized LE on the anti-mesometrial side of the uterine lumen, and syncytializing LE on the mesometrial side of the uterine lumen (Figure 3A). Immunoreactive E-cadherin-positive cells were present within the stroma underlying the syncytializing LE (Figure 3A, the region indicated by the yellow box, shown at higher magnification in the panel to the right), but E-cadherin positive cells were not present in the stroma underlying non-syncytializing LE (Figure 3A, the region indicated by the white box). After laser microdissection, CDH1 (E-cadherin) mRNA was detected by RT-PCR analysis only in stromal tissue that contained E-cadherin-positive cells (Figure 3B,C).

### 2.6. TGCs Invade into the Uterine Stroma and Apoptotic LE Cells Associate with Immune Cells within the Uterine Stroma

**Figure 4:**
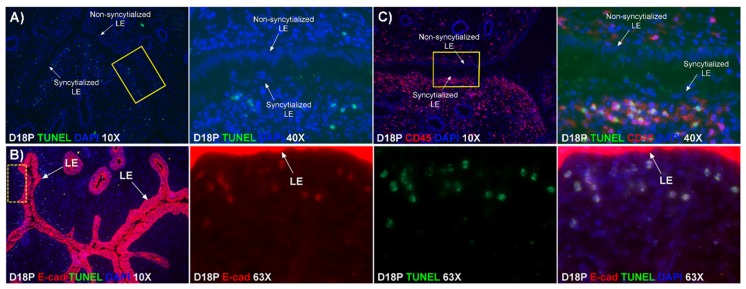
Apoptosis and elimination of LE cells during syncytialization. (**A**) TUNEL staining (green) in the endometrium on day 18 of pregnancy. The second panel represents the region indicated by the yellow box in the first panel. TUNEL-positive cells were detected within the stroma underlying syncytialized LE. (**B**) Double staining for E-cad (red) and TUNEL (green) in the endometrium on day 18 of pregnancy. The second, third, and fourth panels represent the region indicated by the dotted yellow box in the first panel. TUNEL-positive cells co-localized with E-cadherin staining in the stroma. (**C**) Double staining for CD45 (red) and TUNEL (green) at an implantation site. The second panel represents the region indicated by the yellow box in the first panel. CD45-positive cells were accumulated within the stroma underlying syncytialized LE, and these CD45-positive cells were closely associated with TUNEL-positive cells within the stroma. The width of field for the microscopic image captured at 10×, 40×, and 63× is 890, 220, and 140 μm, respectively.

To determine whether the E-cadherin-positive cells within the stroma underlying actively syncytializing LE were undergoing apoptosis, we performed a TUNEL assay that detects cells undergoing DNA fragmentation in the late stage of apoptosis. TUNEL-positive cells were detected within the stroma underlying the syncytializing LE but were barely detectable within stroma underlying intact LE that was not undergoing syncytialization (Figure 4A). The TUNEL positive cells within the stroma were shown to express E-cadherin indicating they were epithelial cells (Figure 4B).

Immunofluorescence staining for CD45, a common marker for leukocytes, showed that leukocytes accumulated within the stroma underlying actively syncytializing LE to a greater extent than in stromal regions underlying intact mononuclear LE cells. Further, these CD45-positive cells were closely associated with TUNEL-positive cells (Figure 4C).

**Figure 5:**
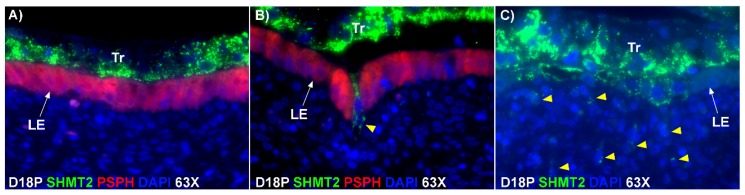
TGCs invade into the uterine stroma during syncytialization. (**A**,**B**) Double immunofluorescence staining for SHMT2 (serine hydroxymethyltransferase 2; green) and PSPH (phosphoserine phosphatase; red) at implantation sites on day 18 of pregnancy. SHMT2-positive trophoblast cells are shown penetrating the PSPH-stained LE layer (yellow arrow head). (**C**) Immunofluorescence staining for SHMT2 at implantation sites on day 18 of pregnancy. SHMT2-positive trophoblast cells were detected within the uterine stroma by day 18 of gestation (yellow arrow heads). Tr, trophoblast cells; LE, luminal epithelium; D, day; P, pregnancy. The width of field for the microscopic images is 140 μm.

Immunofluorescence staining for SHMT2, an enzyme that converts serine to glycine and is expressed by trophoblast cells at the uterine–placental interface of sheep, showed active invasion of SHMT2-positive trophoblast cells through the uterine LE identified by PSPH and into the underlying stroma at sites of syncytialization. SHMT2-expressing cells were present deep within the uterine stroma by day 18 of gestation (Figure 5).

## 3. Discussion

For over 20 years the scientific consensus has been that during trophoblast syncytialization in sheep, BNCs differentiate from the mononuclear trophoblast cells through mitotic polyploidy, individual BNCs fuse with individual LE cells to form trinucleate cells, and BNCs continue to migrate to the LE layer and fuse with these growing trophoblast–LE syncytial plaques. LE cells are incorporated into syncytial plaques, and there is no lateral fusion between syncytial plaques, rather all fusion events involve newly formed BNCs [9,11]. Results of the present study expand our understanding of the syncytialization process in sheep as they indicate that: 1) there are syncytial cells containing more than two nuclei within the trophoblast cell layer, suggesting that fusion between trophectoderm cells is not limited to the formation of BNCs, and that the term ‘trophoblast giant cell (TGC)’ may be appropriate; 2) depolarized uterine LE cells express caspase 3 and stain positively for TUNEL suggesting apoptosis of uterine LE during syncytialization; 3) engulfment of caspase 3-positive uterine LE cells by TGCs and empty spaces/voids within the uterine LE at sites of implantation indicating elimination of apoptotic LE cells by TGCs; 4) rapid enlargement of syncytial plaques suggests that fusion is not limited to the incorporation of new BNCs, but involves lateral fusion between growing syncytial plaques; and 5) E-cadherin- and TUNEL-positive LE cells and SHMT2-positive TGCs within the stroma underlying degenerating LE, coincident with the accumulation of CD45-positive immune cells at these sites suggesting that TGCs carry apoptotic LE cells away from the uterine–placental interface for elimination by immune cells within the uterine stroma.

The mechanism by which BNCs differentiate from mononuclear trophoblasts is poorly understood. BNCs may be generated from mononuclear trophoblast cells by consecutive nuclear divisions without cytokinesis, also termed mitotic polyploidy [8]. Alternatively, the BNCs may form by fusion of mononuclear trophoblast cells [13]. The current consensus for placental syncytialization was based on electron microscopic (EM) studies that observed BNCs in the trophoblast layer and trinucleate cells in the LE layer [12]. With these observations, it is thought that only trophoblast BNCs develop in the trophoblast layer and migrate into the uterine LE, and trinucleate cells in the LE are formed by fusion between BNCs and LE cells (Figure 1D). If this were the case, all PAG-stained trophoblast cells in the trophoblast cell layer should be BNCs. However, results of the present study revealed that multinucleated trophoblast cells with three or four nuclei also migrate into the uterine LE. This suggests that differentiation of trophoblasts is not limited to the formation of BNCs. In addition, the presence of trophoblast cells with three nuclei, an odd number of nuclei that could not be generated by mitotic polyploidy, suggests that fusion of adjacent trophoblast cells may occur to generate multinucleated TGCs that have two or more nuclei. This raises the possibility that at least some of the trinucleate cells that were observed by EM in the LE layer could possibly be TGCs that have three nuclei, entirely of trophoblast origin, that have migrated from the trophoblast layer into the LE layer.

Our results show that extensive migration of TGCs into the LE layer and replacement of mononuclear LE cells by syncytial cells occurs between day 17 and day 20 of pregnancy. An important observation is that there were fewer nuclei in the syncytialized regions of the LE layer as compared to the number of nuclei observed in regions of intact and non-syncytialized LE, suggesting that LE cells may not be incorporated into the syncytial layer during syncytializations, rather there is loss of LE cells during syncytialization. In mice, the uterine LE cells are eliminated during implantation [14]. Based on EM studies, it is hypothesized that degeneration of LE cells is intrinsic to the uterus and embryos play a minor role [15,16], whereas another hypothesis proposed that trophoblast cells trigger apoptosis of LE cells [17]. Regardless of the mechanism involved, most studies found that uterine LE cells adjacent to the blastocyst exhibit characteristics of apoptosis, including cellular shrinkage and nuclear fragmentation, following attachment of blastocysts for implantation and that the apoptotic LE cells are phagocytized by trophoblast cells [17,18,19]. Our data show that LE cells express caspase 3, an executor in the apoptosis cascade, during syncytialization. In addition, TGCs appeared to physically engulf, internalize or fuse with the caspase 3-positive LE cells. At present, the immunofluorescence staining does not discriminate between these three processes. However, taken together, these results suggest that LE cells undergo apoptosis and TGCs associate intimately with these apoptotic LE cells during syncytialization.

E-cadherin, an adherens junction molecule, is specifically expressed in epithelial cells and important for the integrity of epithelial cells. In the present study, E-cadherin-positive cells were detected within the uterine stroma underlying syncytialized regions of the LE layer compared to regions of intact LE. We hypothesize that the E-cadherin-positive cells were LE cells undergoing apoptosis because the localization of E-cadherin-positive cells was limited to the stroma just below the syncytialized LE wherein LE cells appear to undergo apoptosis. Indeed, TUNEL staining co-localized with E-cadherin in those cells. This supports our hypothesis that E-cadherin-positive cells within the stroma are apoptotic LE cells. Further, immunofluorescence staining for CD45, a common marker for leukocytes, showed the accumulation of CD45-positive cells closely associated with TUNEL-positive cells. We hypothesize that these immune cells eliminate the apoptotic LE cells within the stroma. The question arises, how do the LE cells move down to the stroma? We propose that TGCs carry apoptotic LE cells into the stroma, because TGCs appears to be able to engulf LE cells, TGCs are known to be inherently invasive [7,20], and SHMT2-positive trophoblast cells were detected within the uterine stroma underlying regions of active syncytialization. Therefore, it is possible that TGCs engulf or endocytose apoptotic LE cells and then invade the basement membrane cell barrier to carry the LE cells into the underlying stroma where they can be eliminated by immune cells. If the process described above is taking place, then gaps should develop in the LE cell layer where LE cells have been engulfed by TGCs. The results of this study show that gaps in the LE barrier are developed at implantation sites as syncytialization progresses. It is possible that these spaces are replaced by syncytia that likely arose through the lateral fusion of TGCs within the LE cell layer.

**Figure 6:**
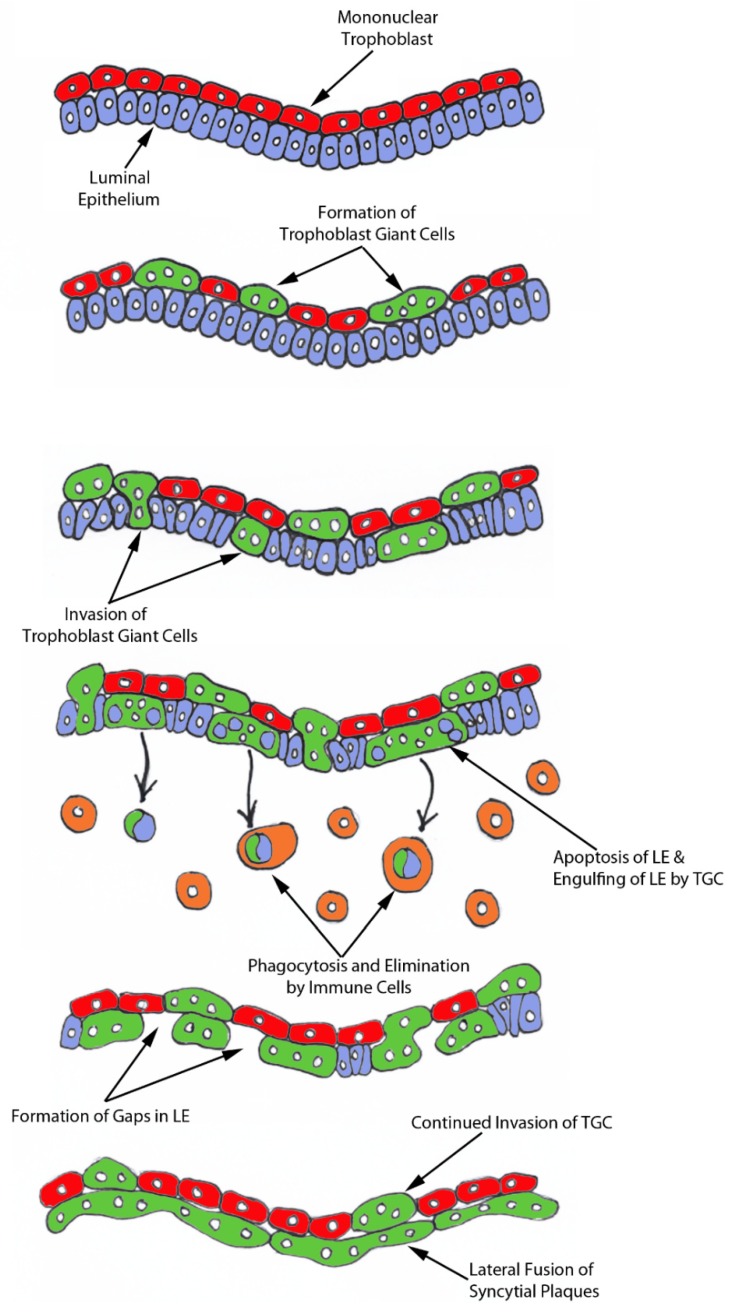
A working hypothesis for the syncytialization of the sheep placenta. Mononuclear trophoblast cells fuse with one-another to become multinucleated TGCs. Large numbers of TGCs migrate to insert themselves between the uterine LE cells that are simultaneously undergoing apoptosis. TGCs then engulf LE cells and carry them to the stroma for elimination by immune cells. The remaining TGCs then fuse with each other to form an extensive trophoblast syncytial layer that fills spaces left by removal of uterine LE and form the interface between caruncles and cotyledons in placentomes of the functional synepitheliochorial placenta of sheep.

In light of our results, we hypothesize that mononuclear trophoblast cells fuse with one-another to become multinucleated TGCs, and a role for the endogenous betaretroviruses (enJSRV) in this process is likely [13,20]. Large numbers of TGCs migrate to insert themselves between the uterine LE cells that are simultaneously undergoing apoptosis. TGCs then engulf LE cells and carry them to the stroma for elimination by immune cells. The remaining TGCs then fuse with each other to form an extensive trophoblast syncytial layer that fills spaces left by removal of uterine LE cells and provides the direct interface between uterine and placental tissues within the placentomes of sheep (Figure 6). Therefore, we suggest that the possibility exists that the composition of the syncytial layer at the uterine–placental interface of sheep is entirely of placental origin. In humans, neighboring cytotrophoblast cells begin to fuse to generate syncytiotrophoblasts at the time of adhesion of the blastocyst to the uterine LE that initiates implantation and this syncytiotrophoblast is the tissue that penetrates through the uterine LE during implantation of the human blastocyst [21]. Our present study suggests that early placentation in sheep is more similar to early placentation in humans than has been understood in that both may develop mononucleated cytotrophoblast and multinucleated syncytiotrophoblast layers of entirely placental origin [22,23].

## 4. Materials and Methods

### 4.1. Animals and Tissue Preparation

Experimental procedures complied with the Guide for Care and Use of Agricultural Animals in Research and Teaching and were approved by the Texas A&M University Institutional Animal Care and Use Committee (AUP IACUC 2012-161). Mature western-range ewes of primarily Suffolk breeding were observed daily for estrous behavior. Following at least two estrous cycles of normal duration (16–18 days), ewes were mated to rams of proven fertility (day 0). Four ewes each were hysterectomized on days 17, 18, 20, and 30 of pregnancy. Several 1–1.5 cm sections of uterine wall from the middle of each horn from pregnant ewes were: (1) embedded in OCT compound, frozen in liquid nitrogen, and stored at −80 °C; and (2) fixed in 4% paraformaldehyde and paraffin-embedded.

### 4.2. Immunofluorescence Analyses

Immunoreactive PAG, E-cadherin, cytokeratin, caspase 3, SHMT2 (serine hydroxymethyltransferase 2), and PSPH (phosphoserine phosphatase) proteins were localized in paraffin-embedded samples from day 17–30 pregnant ewes using immuofluorescence microscopy. Antigen retrieval was performed using either boiling citrate (E-cadherin, cytokeratin, caspase 3, SHMT2, and PSPH) or protease (PAG, E-cadherin, cytokeratin, and caspase 3). Sections were then blocked in 10% normal goat serum for 1 h at room temperature. These sections were incubated overnight at 4 °C with the following primary antibodies: rabbit anti-PAG polyclonal antibody (kindly provided by Jonathan A. Green, University of Missouri-Columbia, Columbia, MO; 1:100), rabbit anti-SHMT2 polyclonal antibody (Sigma-Aldrich, St. Louis, MO, USA; HPA020543; 1:100), rabbit anti-PSPH polyclonal antibody (Lifespan Biosciences, Seattle, WA, USA; LS-B2935; 1:100), mouse anti-E-cadherin monoclonal antibody (BD Biosciences; San Jose, CA, USA; 610182; 1:200), mouse anti-cytokeratin monoclonal antibody (Sigma-Aldrich; C-6909; 1:500), and mouse anti-caspase 3 monoclonal antibody (Cell Signaling Technology; Danvers, MA, USA; 9668; 1:100). Each antibody was used at a dilution optimized for that antibody. Normal rabbit (EMD Millipore; Billerica, MA, USA; 12-370) or mouse (EMD Millipore; 12-371) IgG, at a concentration equal to that for the primary IgG, was used as the negative control. Immunoreactive proteins were detected using the appropriate Alexa Fluor 488- or Alexa Fluor 594-conjugated secondary antibodies (Life Technologies, Grand Island, NY, USA) for 1 h at room temperature at a dilution of 1:250. Tissue sections were then washed three times for 5 min/wash in PBS. Slides were counterstained with Prolong Gold Antifade reagent containing DAPI (Life Technologies, Carlsbad, CA, USA) and coverslipped. Images were taken using an Axioplan 2 microscope (Carl Zeiss, Thornwood, NY, USA) interfaced with an Axioplan HR digital camera.

Frozen sections of uterine–placental interface (8 μm) from day 18 of pregnancy were used to localize CD45- or E-cadherin-positive cells using immunofluorescence microscopy as described previously [24]. Sections were fixed in methanol at −20 °C and washed in PBS. These sections were then blocked in 10% normal goat serum diluted in antibody dilution buffer for 1 h at room temperature. Mouse anti-CD45 monoclonal antibody (Pierce Biotechnology, Rockford, IL, USA; MA1-81267; 1:500) or anti-E-cadherin antibody (1:200) were added at a dilution optimized for each antibody and incubated overnight at 4 °C in a humidified chamber. Tissue sections were then washed three times for 5 min/wash in PBS. Goat anti-mouse IgG Alexa 594 (Life Technologies; 1:250) was added to tissue sections and incubated for 1 h at room temperature. Tissue sections were then washed three times for 5 min/wash in PBS. Slides were processed for counterstaining and imaging as described previously.

For dual immunofluorescence staining (PAG + E-cadherin, PAG + cytokeratin, PAG + caspase 3, SHMT2 + PSPH), we followed the same procedures as described for normal immunofluorescence staining except that we added the two primary antibodies simultaneously on the first day and added the two secondary antibodies (goat anti-rabbit-Alexa Fluor 488-conjugated and goat anti-mouse-Alexa Fluor 594-conjugated) simultaneously on the second day.

### 4.3. TUNEL Apoptosis Assay

Apoptosis was assessed using a terminal deoxynucleotidyl transferase (TdT)-mediated dUTP nick-end labeling (TUNEL) assay (Promega) according to the manufacturer’s instructions for frozen tissue. Briefly, sections were fixed in 4% methanol free paraformaldehyde (PFA) in PBS for 25 min at 4 °C. The tissue was washed with PBS, and then slides were covered with equilibration buffer for 10 min at room temperature followed by incubation with TdT incubation buffer (containing TdT and nucleotide mix) for 1 h at 37 °C in a humidified chamber. The reaction was terminated by submersion of slides in 2× SSC for 15 min at room temperature. The sections were then washed with PBS, counterstained with Prolong Gold Antifade reagent containing DAPI (Life Technologies), and coverslipped. Images were taken using an Axioplan 2 microscope (Carl Zeiss, Thornwood, NY, USA) interfaced with an Axioplan HR digital camera.

For double staining for TUNEL and E-cadherin, mouse anti-E-cadherin antibody was added to TUNEL-stained sections and incubated overnight at 4 °C in a humidified chamber in the dark. Tissue sections were then washed three times for 5 min/wash in PBS. Goat anti-mouse IgG Alexa 594 (Life Technologies; 1:250) was added and incubated for 1 h at room temperature. Tissue sections were then washed three times for 5 min per wash in PBS and processed for imaging as described previously.

### 4.4. Rapid Immunofluorescent Staining and Laser Capture Microdissection (LCM)

Endometrial tissues frozen in OCT were sectioned at 8 μm using a cryostat. Sections were placed on polyethylene naphthalate (PEN) membrane slides and stored at −80 °C until analyzed. Immunofluorescent staining was performed as previously described with slight modifications to maintain mRNA integrity [25]. Briefly, the sections were fixed in ice-cold 100% methanol for 3 min, washed briefly in cold PBS, and incubated with mouse anti-E-cadherin antibody (1:50) for 5 min, and followed by four brief rinses in cold PBS. These sections were then incubated with anti-mouse secondary antibody conjugated to Alexa Fluor 594 for 5 min. Sections were washed four times in cold PBS and dehydrated (1 min each in 75%, 95%, and 2 × 1 min in 100% EtOH), followed by 5 min in xylene. After air-drying for 10 min, sections were visualized using a Veritas™ Laser Microdissection System (Arcturus Bioscience, Mountain View, CA, USA). Stroma containing no E-cadherin-fluorescence-positive cells and stroma containing E-cadherin-fluorescence-positive cells were collected on the CapSure Macro LCM Caps (Thermo Fisher Scientific, Waltham, MA, USA).

### 4.5. Reverse Transcription–Polymerase Chain Reaction (RT–PCR)

Total RNA was prepared from captured cells using a PicoPure RNA Isolation Kit (Thermo Fisher Scientific), and cDNA was synthesized using a Superscript III First Strand Kit (Life Technologies, Carlsbad, CA, USA) according to the manufacturer’s instructions. The cDNA templates were amplified by PCR using Taq polymerase and specific primers based on mRNA sequences of sheep CDH1 (GenBank accession number XM_004015093.3; forward, 5′-TAC TAT GAT GAA GAA GGA GGT GGA G-3′; reverse, 5′-CAA TAA AGT TTC CAA TTT CAT CAG G-3′) or ACTB (GenBank accession number NM_001009784.1; forward, 5′-ATT CAC GAA ACT ACC TTC AAT TCC-3′; reverse, 5′-ATG ATC TTG ATC TTC ATC GTG CT-3′), which amplify cDNAs of 175 bp and 170 bp, respectively. PCR conditions for both CDH1 and ACTB were 45 cycles of 94 °C for 45 s, 60 °C for 45 s, and 72 °C for 1 min. PCR products were separated on 2% agarose gels and visualized following ethidium bromide staining.

## Supplementary Materials

Supplementary materials can be found at https://www.mdpi.com/1422-0067/20/18/4530/s1.

## References

[1] Wintenberger-Torres S., Flechon J.E. (1974). Ultrastructural evolution of the trophoblast cells of the pre-implantation sheep blastocyst from day 8 to day 18. J. Anat..

[2] Rowson L.E., Moor R.M. (1966). Development of the sheep conceptus during the first fourteen days. J. Anat..

[3] Farin C.E., Imakawa K., Roberts R.M. (1989). In situ localization of mRNA for the interferon, ovine trophoblast protein-1, during early embryonic development of the sheep. Mol. Endocrinol..

[4] Guillomot M., Michel C., Gaye P., Charlier N., Trojan J., Martal J. (1990). Cellular localization of an embryonic interferon, ovine trophoblastin and its mRNA in sheep embryos during early pregnancy. Biol. Cell.

[5] Godkin J.D., Bazer F.W., Thatcher W.W., Roberts R.M. (1984). Proteins released by cultured day 15–16 conceptuses prolong luteal maintenance when introduced into the uterine lumen of cyclic ewes. J. Reprod. Fertil..

[6] Wimsatt W.A. (1951). Observations on the morphogenesis, cytochemistry, and significance of the binucleate giant cells of the placenta of ruminants. Am. J Anat..

[7] Hoffman L.H., Wooding F.B. (1993). Giant and binucleate trophoblast cells of mammals. J Exp. Zool..

[8] Klisch K., Hecht W., Pfarrer C., Schuler G., Hoffmann B., Leiser R. (1999). DNA content and ploidy level of bovine placentomal trophoblast giant cells. Placenta.

[9] Wooding F.B., Wathes D.C. (1980). Binucleate cell migration in the bovine placentome. J. Reprod. Fertil..

[10] Wooding F.B. (1982). The role of the binucleate cell in ruminant placental structure. J. Reprod. Fertil. Suppl..

[11] Wooding F.B. (1992). Current topic: The synepitheliochorial placenta of ruminants: Binucleate cell fusions and hormone production. Placenta.

[12] Wooding F.B.P., Burton G., Wooding F.B.P., Burton G. (2008). Synepitheliochorial placentation: Ruminants (ewe and cow). Comparative Placentation: Structures, Functions and Evolution.

[13] Dunlap K.A., Palmarini M., Spencer T.E. (2006). Ovine endogenous betaretroviruses (enJSRVs) and placental morphogenesis. Placenta.

[14] Li Y., Sun X., Dey S.K. (2015). Entosis allows timely elimination of the luminal epithelial barrier for embryo implantation. Cell Rep..

[15] Finn C.A., Hinchliffe J.R. (1964). Reaction of the mouse uterus during implantation and deciduoma formation as demonstrated by changes in the distribution of alkaline phosphatase. J. Reprod. Fertil..

[16] Krehbiel R.H. (1937). Cytological Studies of the Decidual Reaction in the Rat during Early Pregnancy and in the Production of Deciduomata. Physiol. Zool..

[17] Parr E.L., Tung H.N., Parr M.B. (1987). Apoptosis as the mode of uterine epithelial cell death during embryo implantation in mice and rats. Biol. Reprod..

[18] Welsh A.O., Enders A.C. (1993). Chorioallantoic placenta formation in the rat. III. Granulated cells invade the uterine luminal epithelium at the time of epithelial cell death. Biol. Reprod..

[19] El-Shershaby A.M., Hinchliffe J.R. (1975). Epithelial autolysis during implantation of the mouse blastocyst: An ultrastructural study. J. Embryol. Exp. Morphol..

[20] Spencer T.E., Johnson G.A., Bazer F.W., Burghardt R.C., Palmarini M. (2007). Pregnancy recognition and conceptus implantation in domestic ruminants: Roles of progesterone, interferons and endogenous retroviruses. Reprod. Fertil. Dev..

[21] Huppertz B., Gauster M. (2011). Trophoblast fusion. Adv. Exp. Med. Biol..

[22] Midgley A.R., Pierce G.B., Deneau G.A., Gosling J.R. (1963). Morphogenesis of syncytiotrophoblast in vivo: An autoradiographic demonstration. Science.

[23] Panigel M., Redman C., Sargent I., Starkey P.M. (1993). The origin and structure of extraembryonic tissues. The human placenta.

[24] Burghardt R.C., Burghardt J.R., Taylor II J.D., Reeder A.T., Nguen B.T., Spencer T.E., Bayless K.J., Johnson G.A. (2009). Enhanced focal adhesion assembly reflects increased mechanosensation and mechanotransduction at maternal-conceptus interface and uterine wall during ovine pregnancy. Reproduction.

[25] Williams D.L., Schwartz M.W., Bastian L.S., Blevins J.E., Baskin D.G. (2008). Immunocytochemistry and laser capture microdissection for real-time quantitative PCR identify hindbrain neurons activated by interaction between leptin and cholecystokinin. J. Histochem. Cytochem..

