# Supplementary figures and images for: Immunohistochemical Examination of Trophoblast Syncytialization during Early Placentation in Sheep
Source: Int J Mol Sci. 2019 Sep 13;20(18):4530. doi: 10.3390/ijms20184530 (PMC6769582; doi:10.3390/ijms20184530)

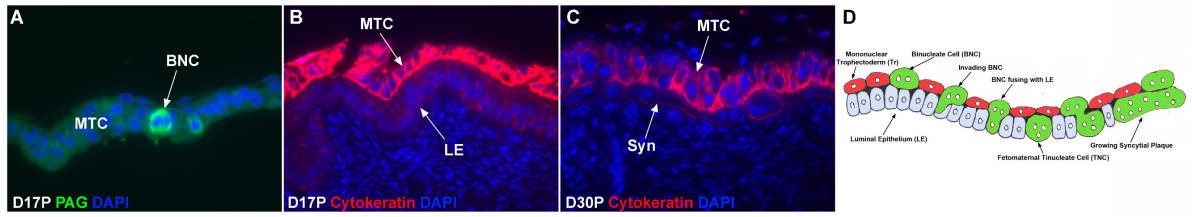

Supplement: Supplementary file 1 [file ijms-20-04530-s001.pdf]
